# YOLOv8-RMDA: Lightweight YOLOv8 Network for Early Detection of Small Target Diseases in Tea

**DOI:** 10.3390/s24092896

**Published:** 2024-05-01

**Authors:** Rong Ye, Guoqi Shao, Yun He, Quan Gao, Tong Li

**Affiliations:** 1 College of Food Science and Technology, Yunnan Agricultural University, Kunming 650201, China; 15912913557@163.com; 2 The Key Laboratory for Crop Production and Smart Agriculture of Yunnan Province, Kunming 650201, China; 15751769522@163.com; 3 Big Data College, Yunnan Agricultural University, Kunming 650201, China; heyun@ynau.edu.cn (Y.H.); gaoq@ynau.edu.cn (Q.G.)

**Keywords:** YOLOv8, tea leaf spot detection, inner-EIoU, AKConv, dynamic head

## Abstract

In order to efficiently identify early tea diseases, an improved YOLOv8 lesion detection method is proposed to address the challenges posed by the complex background of tea diseases, difficulty in detecting small lesions, and low recognition rate of similar phenotypic symptoms. This method focuses on detecting tea leaf blight, tea white spot, tea sooty leaf disease, and tea ring spot as the research objects. This paper presents an enhancement to the YOLOv8 network framework by introducing the Receptive Field Concentration-Based Attention Module (RFCBAM) into the backbone network to replace C2f, thereby improving feature extraction capabilities. Additionally, a mixed pooling module (Mixed Pooling SPPF, MixSPPF) is proposed to enhance information blending between features at different levels. In the neck network, the RepGFPN module replaces the C2f module to further enhance feature extraction. The Dynamic Head module is embedded in the detection head part, applying multiple attention mechanisms to improve multi-scale spatial location and multi-task perception capabilities. The inner-IoU loss function is used to replace the original CIoU, improving learning ability for small lesion samples. Furthermore, the AKConv block replaces the traditional convolution Conv block to allow for the arbitrary sampling of targets of various sizes, reducing model parameters and enhancing disease detection. the experimental results using a self-built dataset demonstrate that the enhanced YOLOv8-RMDA exhibits superior detection capabilities in detecting small target disease areas, achieving an average accuracy of 93.04% in identifying early tea lesions. When compared to Faster R-CNN, MobileNetV2, and SSD, the average precision rates of YOLOv5, YOLOv7, and YOLOv8 have shown improvements of 20.41%, 17.92%, 12.18%, 12.18%, 10.85%, 7.32%, and 5.97%, respectively. Additionally, the recall rate (R) has increased by 15.25% compared to the lowest-performing Faster R-CNN model and by 8.15% compared to the top-performing YOLOv8 model. With an FPS of 132, YOLOv8-RMDA meets the requirements for real-time detection, enabling the swift and accurate identification of early tea diseases. This advancement presents a valuable approach for enhancing the ecological tea industry in Yunnan, ensuring its healthy development.

## 1. Introduction

In the current era of global competition, the significance of agriculture cannot be understated. Tea, as a vital cash crop in my country, plays a crucial role in securing economic stability for tea farmers within the framework of the rural revitalization policy. Being a specialty industry in Yunnan Province and a renowned ‘golden brand’ in regional agriculture, the high-quality growth of the tea sector can substantially bolster the competitive edge of regional specialty industries [1].

Unfavorable environmental conditions such as strong light radiation, low night temperatures, and high daytime humidity in Yunnan can lead to diseases that affect the yield and quality of tea plants. Disease stands out as the primary factor that hinders the stable growth of plant yield and quality. Severe cases of disease outbreaks can have catastrophic effects on smallholder economies heavily reliant on agriculture. As the variety of tea types increases, planting areas expand, and cultivation methods evolve, numerous secondary diseases have started to surface. Detecting diseases early on, gathering disease information promptly, identifying infection causes accurately, and assessing disease severity are crucial steps that can help reduce pesticide usage, minimize environmental pollution, and effectively prevent and control diseases before they spread and lead to yield decline. Therefore, the early diagnosis and identification of diseases in tea gardens play a vital role in ensuring high and efficient tea production [2].

In recent years, deep learning has made significant strides in various fields, including agriculture, thanks to advancements in data analysis and image-processing technology. Target detection using deep learning has emerged as a key area of research in computer vision, with applications in crop maturity detection [3,4], pest and disease identification [5,6,7,8,9,10], plant phenotyping [11,12,13], and weed management [14,15]. Through the development of sophisticated parallel models, challenges such as scattered data resources, information integration complexities, and inefficient knowledge utilization in agricultural settings have been effectively addressed.

Currently, popular deep learning target detection models such as Faster R-CNN [16,17], SSD [18,19], and the YOLO [20,21,22,23,24,25] series are widely used in various research studies. Researchers are continuously enhancing and refining these models for applications in crop disease classification and detection. For instance, Li et al. [26] employed YOLOv5n to identify cucumber diseases, achieving improved accuracy through the incorporation of a coordinated attention mechanism and transformer structure. Sun Fenggang et al. [27] utilized an enhanced version of YOLOv5s for the rapid detection of apple fruit diseases. Xue et al. [28] introduced YOLO-Tea, a model for detecting tea pests and diseases based on an enhanced YOLOv5, which significantly enhanced the accuracy and speed in identifying tea leaf diseases and pests in natural settings. Additionally, Fuentes et al. [29] investigated tomato pests and diseases using various deep learning architectures and feature extraction methods for designing detection networks. Zhou et al. [30] applied the YOLOv7 algorithm and image-processing techniques to locate and extract the center point of Camellia oleifera fruit, achieving an average accuracy of 94.7%, surpassing the YOLOv5s algorithm by 0.7 percentage points.

Current mainstream target detection frameworks often do not include specific enhancements for small targets. When the targets are smaller in size, existing target detection algorithms exhibit a noticeable decrease in performance. The overall performance is affected as follows:

1. When the detection target is small and dense, as the network deepens during the training process, the detected objects may lose edge information, grayscale information, and other features. This can lead to the mixing of irrelevant features during model training, along with a significant amount of image noise information, ultimately reducing model accuracy.
2. The size of the receptive field mapped to the original image is a key factor in the success of target detection. A small receptive field preserves spatial structural features while potentially compromising abstract semantic information. Conversely, a large receptive field retains rich semantic information but may lose spatial structure details of the target. Many methods aim to enhance the recognition accuracy of crop diseases by boosting network complexity yet fail to fundamentally improve features. This results in information loss, redundancy during extraction, increased hardware resource consumption, and reduced recognition speed.

In order to realize the combination of computer vision technology and the dynamic recognition of tea diseases, based on the YOLOv8 network framework, this paper introduces the Receptive Field Concentration-Based Attention Module into the backbone network. The RFCBAM replaces C2f in the backbone and enhances the feature extraction capability of the backbone network. The Mixed Pooling SPPF (MixSPPF) module is proposed to increase information blending between features of different levels. The efficient RepGFPN module is introduced to further improve the feature extraction capability of the disease target. The Dynamic Head module is embedded in the detection head part, the multi-attention mechanism is applied to detect the multi-scale, spatial position, and multi-task perception ability of the head-strengthening algorithm, and the inner-IoU loss function is used to improve the EIoU loss function, replacing the original CIoU with inner-EIoU to improve the learning ability of the small lesion samples. In addition, traditional Conv blocks are replaced with AKConv blocks to complete the arbitrary sampling of a variety of targets of different sizes, reducing model parameters and adding momentum to disease detection.

## 2. Related Work

### 2.1. Introduction to the YOLOv8 Algorithm

YOLOv8 is a SOTA (state-of-the-art) model developed by Ultralytics in January 2023, inheriting the strengths of the YOLO series while adding new features and improvements, and consists of three main components: backbone, neck, and head [31].

The backbone component focuses on feature extraction by incorporating the C2f (CSPLayer_2Conv) module for residual learning, inspired by the CSP and ELAN methodologies. It utilizes jump-layer connections and additional split operations to effectively integrate gradient changes into the feature map throughout the process. The Conv convolution module and C2f module are stacked four times in series, with each stack referred to as a stage. The SPPF (Spatial Pyramid Pooling Fusion) module is then employed to standardize the vector sizes of feature maps across various scales. The neck component primarily handles feature fusion, replacing the C3 module with the C2f module and leveraging concepts from PANs (Path Aggregation Networks) and FPNs (Feature Pyramid Networks) to establish top-down and bottom-up feature pyramids. Subsequently, the output features from different stages of the backbone are upsampled directly. The head component enhances the original anchor-based coupling head of YOLOv5 by transitioning to the anchor-free decoupling head, eliminating the objectness branch. Additionally, it features three detection heads with varying size feature maps to identify and output target objects of different sizes.

### 2.2. Improved YOLOv8s Overall Structure

**Figure 1:**
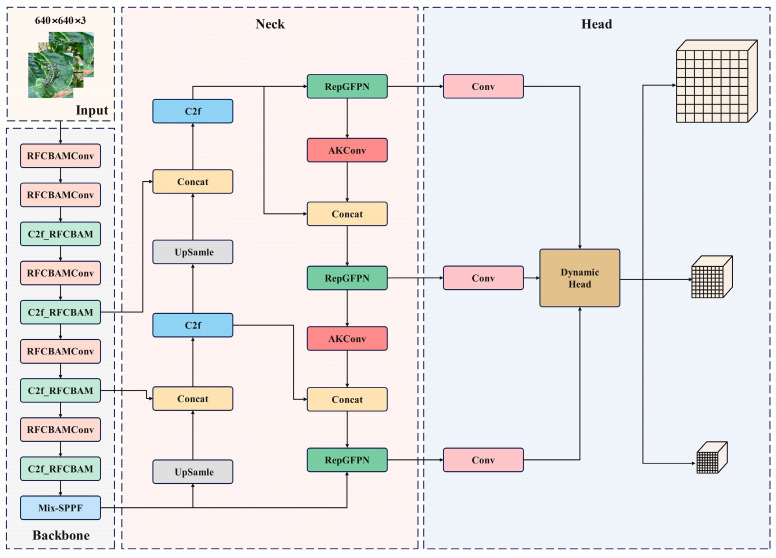
Structure of improved YOLOv8s network.

The enhancement and application of deep learning networks have practical importance for detection tasks in various intricate practical environments [32]. This study introduces enhancements based on YOLOv8 to effectively detect small targets of early tea diseases in complex scenarios. The modified structure is illustrated in Figure 1.

#### 2.2.1. Backbone Network Improvements

**Figure 2:**
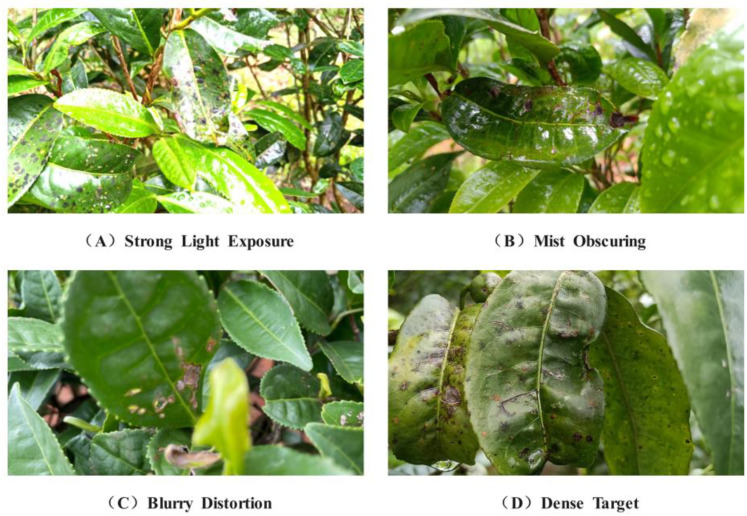
Disease image data sample.

Yunnan boasts a favorable climate and soil, along with a picturesque ecological setting. Tea plants thrive in mountainous regions at altitudes ranging from 2000 to 2500 m, where the peaks are enveloped in clouds and mist. Various factors such as weather fluctuations, changes in light radiation, water mist obstruction, and water vapor generation can lead to visual disturbances, as illustrated in Figure 2.

**Figure 3:**
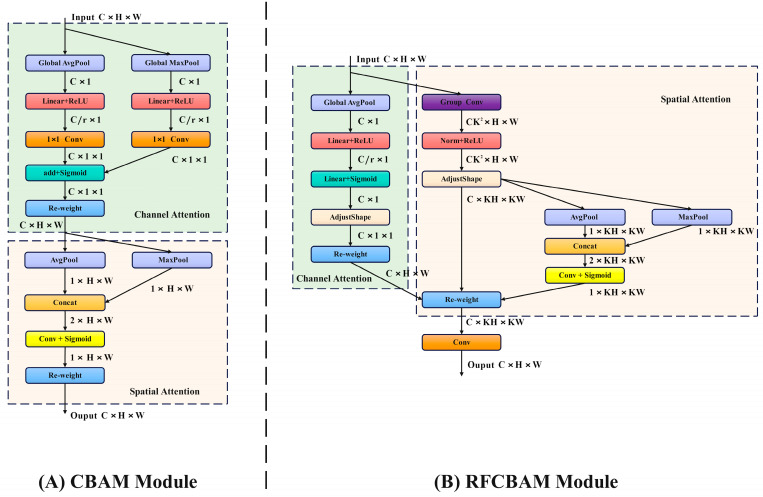
Structure comparison diagram of CBAM and RFCBAM.

The backbone layer is known to introduce significant noise while extracting image features from complex scenes. This noise can disrupt the long-range dependencies between pixels, diminish the model’s ability to detect and recognize objects, and potentially result in false or missed detections. To address this issue, this study enhances the C2f module within the backbone layer. By emphasizing spatial attention features and directing focus towards receptive field spatial features, the study aims to mitigate the impact of noise on the model. This approach resolves the challenge of convolution kernel parameter sharing, ultimately enhancing model performance. The spatial attention mechanism in CBAM is leveraged to target receptive field spatial features, resulting in the development of the RFCBAM (Receptive Field Concentration-Based Attention Module). This module enables the model to capture long-range information dependencies akin to self-attention mechanisms, thereby boosting convolution performance. Figure 3 illustrates the improved RFCBAM. Unlike traditional approaches that treat channel and spatial attention separately, this module integrates both aspects simultaneously. Furthermore, to streamline computational processes, grouped convolution is employed to extract receptive field spatial features and minimize feature redundancy.

**Figure 4:**
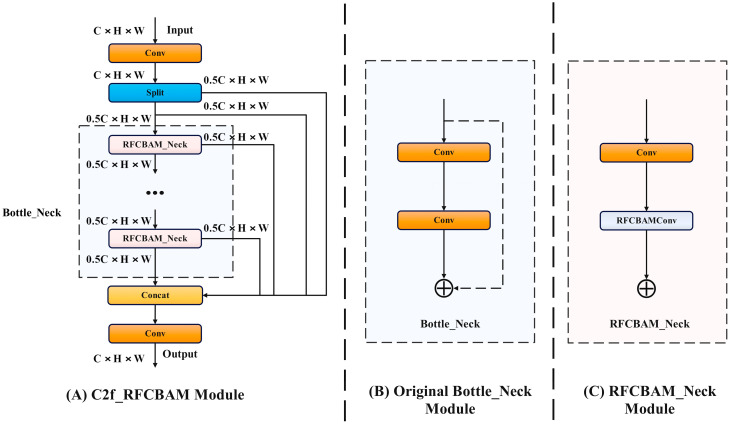
Structure of C2f_RFCBAM.

RFCBAM and C2f_RFCBAMF are utilized to replace the Conv and C2f convolutions in the backbone section of the original YOLOv8 model. This replacement not only enhances the spatial features with improved receptive field attention, but also boosts channel attention, thereby improving feature extraction in both spatial and channel dimensions. By applying the RFCBAM to transform the bottleneck of the C2f module in the backbone, more refined feature information can be obtained. The specific structural modifications are illustrated in Figure 4. The C2f module, enhanced by the RFCBAM, primarily incorporates RFCBAM_Neck to substitute the bottleneck in the original module. Within RFCBAM_Neck, two convolution modules are employed, and the second Conv is replaced with RFCAMConv to eliminate residual connections in RFCBAM_Neck.

#### 2.2.2. MixSPPF Module

**Figure 5:**
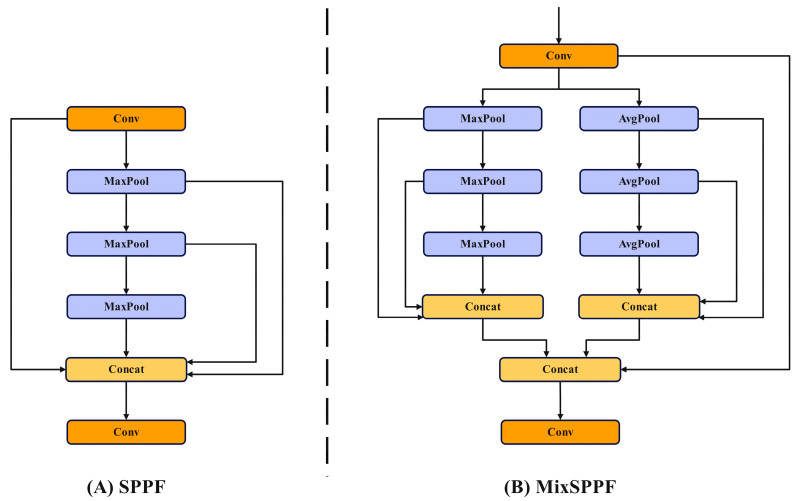
The structure of MixSPPF.

SPPF uses three max pooling methods to extract input features in series. However, max pooling only extracts the maximum value of the input feature and can only represent the local information of the input feature, ignoring the global feature information of the input image. Therefore, this paper uses MixSPPF, and a combination of average pooling and maximum pooling is used to improve the extraction of global information by SPPF. The network structure is shown in Figure 5. Figure 5A shows the SPPF network structure, and Figure 5B shows the MixSPPF structure.

Compared with SPPF, MixSPPF incorporates an average pooling branch that connects three average pooling branches in series. The final output of MixSPPF is obtained by concatenating the outputs of three maximum pooling operations and three average pooling operations. The calculation process is detailed in Formulas (1)–(4).

(1) $$x=Conv(x_{input})$$

(2) $$y_{max}=Cat\left(Max\left(Max(Max(x))\right),Max(Max(x)),Max(x)\right)$$

(3) $$y_{avg}=Cat\left(Avg\left(Avg(Avg(x))\right),Avg(Avg(x)),Avg(x)\right)$$

(4) $$y_{out}=Conv(Cat(y_{max},y_{min}))$$

In the formula, $x_{input}$ represents the input feature; Conv represents the convolution operation; Max represents the maximum pooling operation; Avg represents the average pooling operation; Cat represents the feature splicing operation; $y_{max}$ represents the output feature of the maximum pooling branch; $y_{avg}$ represents the output features of the average pooling branch; $y_{out}$ represents the final output features.

#### 2.2.3. Dynamic Head

**Figure 6:**
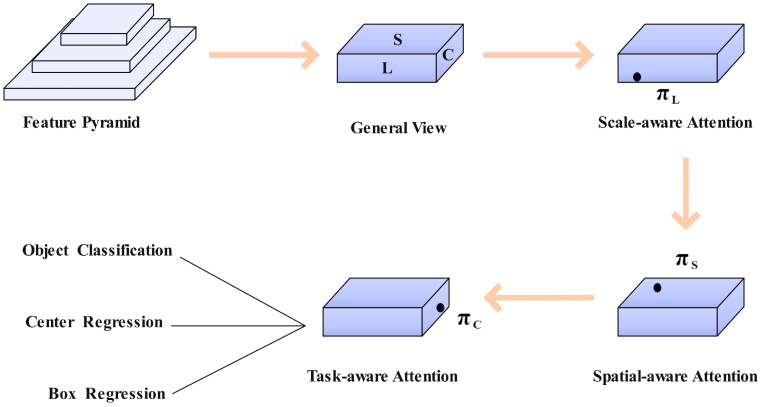
Dynamic Head overall structure diagram.

When capturing images of diseases, especially in harsh climate conditions, there is a risk of losing important pixel information of the disease target. Most current algorithms focus on enhancing the performance of the detection head to identify the target from a consistent viewpoint. In order to enhance the ability to extract crucial features of lesions, this study introduces a novel method called Dynamic Head, which incorporates multiple dynamic attention mechanisms. These attention mechanisms, focusing on scale perception, spatial position, and multi-tasking, aim to enhance the expression capability of the model’s target detection head. This, in turn, improves the model’s accuracy in recognizing various disease targets within complex backgrounds. The structure of Dynamic Head is illustrated in Figure 6.

The feature pyramid is extracted using the backbone network and then adjusted to a three-dimensional feature vector of the same scale. Subsequently, the dynamic detection head is inputted, leading to the output of classification detection for multiple diseases. Formula (5) is as follows:

(5) $$F\in R^{L\times S\times C}$$

In the dynamic detection head framework, the input of the head part is regarded as a three-dimensional level∗space∗channel. Here, level refers to the feature level, space represents the product of width and height of the feature map (H×W), and channel denotes the number of channels in mathematical terms. As a result, self-attention is typically formulated as shown in Formula (6).

(6) W(F)=π(F)⋅F

In the formula, π(⋅) represents the attention function. This type of attention is typically implemented using a fully connected layer, which can result in a sudden surge in computational load and is not conducive to high-dimensional calculations. In contrast, Dynamic Head converts the attention function into three consecutive attentions, each of which only needs to focus on one dimension:

(7) $$\left.W\left(F\right)=\pi_{C}\left(\pi_{S}\left(\pi_{L}\left(F\right)\cdot F\right)\cdot F\right)\cdot F\right)$$

The formula includes three attention functions, $\pi_{L}$, $\pi_{S}$, and $\pi_{C}$, applied to dimensions L, S, and C. The scale-aware attention module $\pi_{L}$ combines features of varying scales according to their semantic significance.

(8) $$\pi_{L}\left(F\right)\cdot F=\sigma (f(\frac{1}{SC}\sum_{S,C}\;F))\cdot F$$

In the formula, f(⋅) represents a linear function approximated using 1*1 convolution. $\sigma (x)=max(0,min(1,\frac{x+1}{2}))$ is a hard sigmoid function.

The spatial perception attention module, denoted as $\pi_{S}$, is dedicated to enhancing the discriminative ability across various spatial locations. Due to the high latitude of *S*, it is essential to decouple the module to facilitate sparse attention learning within the same space. This enables the aggregation of cross-level features at specific locations.

(9) $$\pi_{S}\left(F\right)\cdot F=\frac{1}{L}\sum_{l=1}^{L}\;\sum_{k=1}^{K}\;w_{l,k}\cdot F\left(l;p_{k}+\Delta p_{k};c\right)\cdot \Delta m_{k}$$

In the formula, w represents the variable convolution layer weight; K denotes the number of sparse sampling positions; $p_{k}$ stands for the convolution center point; $\Delta p_{k}$ indicates the relative center point offset; $p_{k}+\Delta p_{k}$ focuses on the judgment area; $\Delta m_{k}$ relates to the weight measurement factor around the position $p_{k}$, which can be acquired from the input features of the intermediate level of F.

The multi-task attention module utilizes the Dynamic ReLU function to activate the input feature map on a per-channel basis. The calculation formula for this activation is:

(10) $$\pi_{C}(F)\cdot F=max(\alpha^{1}(F)\cdot F_{C}+\beta^{1}(F),\alpha^{2}(F)\cdot F_{C}+\beta^{2}(F))$$

In the formula, $F_{C}$ represents the feature slice of the C-th channel, and ${\left.\left[\begin{array}{cccc} \alpha^{1}, & \alpha^{2}, & \beta^{1}, & \beta^{2} \end{array}\right.\right]}^{T}=\theta (\cdot )$ is the learning control activation threshold super function.

**Figure 7:**
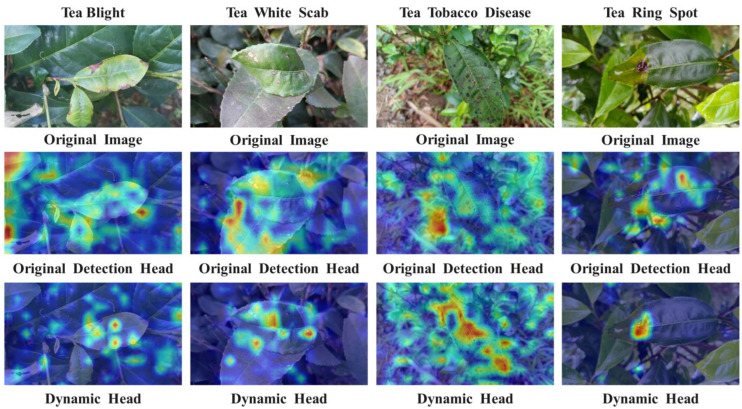
Comparison of heat maps before and after the introduction of Dynamic Head.

To better illustrate the impact of the Dynamic Head target detection head on various tea leaf lesion targets amidst complex backgrounds, Grad-CAM heat map visualization is employed for analysis, heat maps are mainly used to display the location and confidence of target objects in various areas in the image. The darker the area, the higher the probability that the model believes that there is a target object in the area, as depicted in Figure 7. The results demonstrate that the inclusion of Dynamic Head for the four diseases of tea leaf blight, tea white spot, tea coal leaf disease, and tea ring spot enhances the detection head’s capability to accurately locate disease targets, thereby improving model accuracy significantly.

#### 2.2.4. *Inner-IoU*

The bounding box regression (BBR) loss function is continuously updated and optimized with the rapid development of detectors. However, the current IoU-based BBR mainly focuses on accelerating convergence by introducing new loss terms, while overlooking the inherent limitations of the *IoU* loss term itself. Enhancing the *IoU* loss term can partially compensate for the deficiencies of bounding box regression, but it lacks the ability to adapt autonomously to different detectors and detection tasks in practical scenarios. For instance, in the context of detecting tea diseases, where most diseases manifest as densely growing lesions, it becomes crucial for the model to consider various metrics in the bounding box regression, such as distance, overlap area, aspect ratio, etc., between the predicted box and the ground truth box. *IoU*, a key component of the predominant bounding box regression loss function, is defined as follows:

(11) $$IoU=\frac{\left|B\cap B^{gt}\right|}{\left|B\cup B^{gt}\right|}$$

In the formula, B and $B^{gt}$ represent the prediction box and GT box, respectively. After defining *IoU*, its corresponding loss can be defined as:

(12) $$L_{IoU}=1-IoU$$

Existing methods primarily rely on *IoU* and incorporate additional loss terms. *GIoU* addresses the issue of gradient disappearance that occurs when the overlapping area between the anchor box and the GT box is 0. The definition of GIoU is presented in Equation (13):

(13) $$L_{GIoU}=1-IoU+\frac{\left|C-B\cap B^{gt}\right|}{\left|C\right|}$$

In the formula, C is the smallest box covering B and $B^{gt}$.

Compared with *GIoU*, *DIoU* adds a new distance loss term based on *IoU* by minimizing the normalized distance between the center points of the two bounding boxes. The definition is as follows:

(14) $$L_{DIoU}=1-IoU+\frac{\rho^{2}\left(b,b^{gt}\right)}{c^{2}}$$

In the formula, b and $b^{gt}$ represent the center points of B and $B^{gt}$, respectively. The function ρ(⋅) denotes the Euclidean distance, and c stands for the diagonal of the minimum bounding box. *CIoU* [33,34] extends this by incorporating shape loss and introducing a shape loss term derived from *DIoU* loss. The definition can be summarized as follows:

(15) $$L_{CIoU}=1-IoU+\frac{\rho^{2}\left(b,b^{gt}\right)}{c^{2}}+\alpha v$$

(16) $$\alpha =\frac{v}{\left(1-IoU\right)+v}$$

(17) $$v=\frac{4}{\pi^{2}}(arctan\frac{w^{gt}}{h^{gt}}-arctan\frac{w}{h}{)}^{2}$$

In the formula, v represents the consistency of the aspect ratio; α is a positive weight parameter; $w^{gt}$ and $h^{gt}$ represent the width and height of the target frame; w and h represent the width and height of the prediction frame.

Compared with *DIoU*, *EIoU* calculates the normalized difference between the width (w, $w^{gt}$), height (h, $h^{gt}$), and center position (b, $b^{gt}$) of the target box and the anchor box directly, based on *DIoU*. The definition is as follows:

(18) $$L_{EIoU}=1-IoU+\frac{\rho^{2}\left(b,b^{gt}\right)}{c^{2}}+\frac{\rho^{2}\left(w,w^{gt}\right)}{(w^{c}{)}^{2}}+\frac{\rho^{2}\left(h,h^{gt}\right)}{(h^{c}{)}^{2}}$$

In the formula, $w^{c}$ and $h^{c}$ are the width and height of the minimum bounding box of the target box and prediction box, respectively.

**Figure 8:**
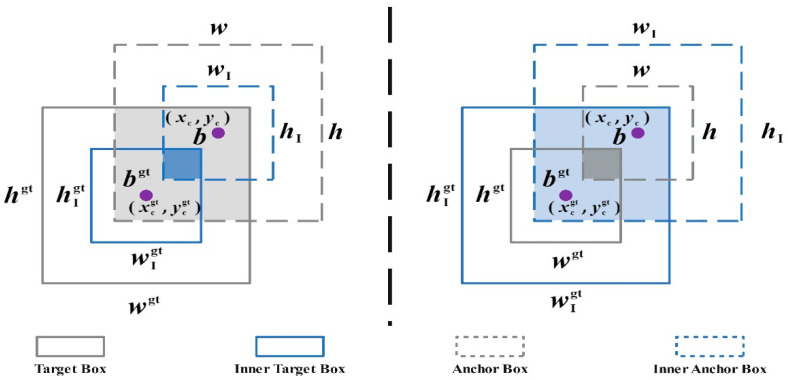
*Inner-IoU* example structure.

In this study, the *EIoU* loss function is utilized. Considering the limitations of the *IoU* loss in terms of rationality and convergence speed, the *inner-IoU* loss is incorporated along with auxiliary bounding boxes to expedite regression without introducing additional loss terms. The *inner-IoU* loss introduces a scale factor ratio to regulate the size of the auxiliary bounding box. This concept is visually represented in Figure 8.

(19) $$b_{l}^{gt}=x_{c}^{gt}-\frac{w^{gt}*ratio}{2},b_{r}^{gt}=x_{c}^{gt}+\frac{w^{gt}*ratio}{2}$$

(20) $$b_{t}^{gt}=y_{c}^{gt}-\frac{h^{gt}*ratio}{2},b_{b}^{gt}=y_{c}^{gt}+\frac{h^{gt}*ratio}{2}$$

(21) $$b_{l}=x_{c}-\frac{w*ratio}{2},b_{r}=x_{c}+\frac{w*ratio}{2}$$

(22) $$b_{t}=y_{c}-\frac{h*ratio}{2},b_{b}=y_{c}+\frac{h*ratio}{2}$$

(23) $$inter=(min(b_{r}^{gt},b_{r})-max(b_{l}^{gt},b_{l}))*(min(b_{b}^{gt},b_{b})-max(b_{t}^{gt},b_{t}))$$

(24) $$union=(w^{gt}*h^{gt})*(ratio{)}^{2}+(w*h)*(ratio{)}^{2}-inter$$

(25) $$IoU^{inner}=\frac{inter}{union}$$

In the formula, ($x_{c}^{gt}$, $y_{c}^{gt}$) represents the center point of the target frame; ($x_{c}$, $y_{c}$) represents the center point of the anchor frame; $w^{gt}$ and $h^{gt}$ represent the width and height of the target frame; w and h represent the anchor frame width and height; ratio is an adjustable scaling factor, with a value range of [0.5, 1.5]. Similar to *IoU* loss, the value range of *inner-IoU* loss is [0, 1]. In comparison to *IoU* loss, *inner-IoU* loss is more effective when the *ratio* is less than 1. When the size of the auxiliary bounding box is smaller than the actual bounding box, the regression’s effective range is smaller than *IoU* loss, but the gradient’s absolute value is larger, leading to faster convergence of high-*IoU* samples. Applying *inner-IoU* loss to *EIoU* results in $L_{Inner-EIoU}$:

(26) $$L_{Inner-EIoU}=L_{EIoU}+IoU-IoU^{inner}$$

#### 2.2.5. AKConv

**Figure 9:**
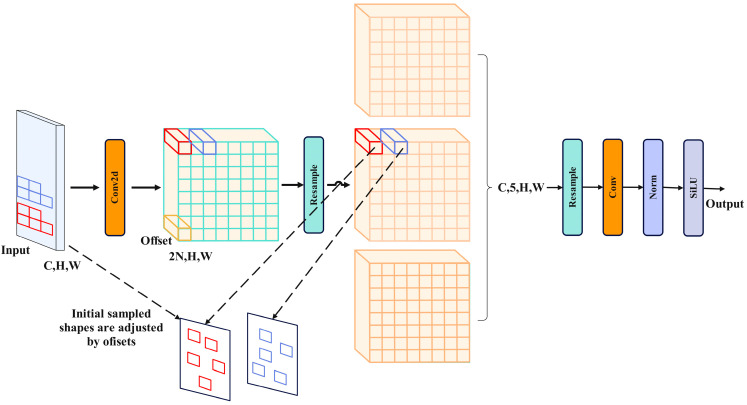
AKConv structure.

Current neural networks utilizing convolution operations have shown impressive advancements in the realm of deep learning [35,36]. However, traditional convolution operations still face limitations. Firstly, the sampling shape size is fixed, restricting the convolution operation to a local window and hindering the capture of information from other locations. Secondly, the convolution kernel size is fixed to a k × k square, leading to exponential growth in parameter computation as the size increases, making lightweight model construction challenging. To address these issues, this study introduces variable kernel convolution (AKConv), which allows for any number of parameters and sampling shapes for the convolution kernel. This not only enhances model performance but also reduces the number of model parameters. The structure is depicted in Figure 9 below.

In AKConv, the input image dimension is set to (C, H, W), where C represents the number of channels, and H and W represent the height and width of the image. The convolution operation begins by applying the initial sampling shape of the convolution kernel to the input image using Conv2d. Subsequently, the initial sampling shape is adjusted through learned offsets, a crucial step in AKConv that enables the dynamic adaptation of the convolution kernel shape to the image’s characteristics. Following this adjustment, AKConv resamples the feature map based on the modified sampling shape. The resampled feature map undergoes reshaping, convolution, normalization, and activation through the SiLU function to produce the final output.

**Figure 10:**
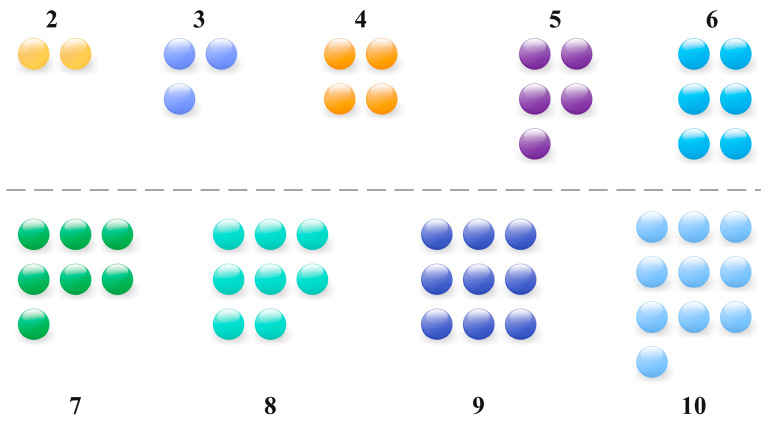
Initial sampling shape.

**Figure 11:**
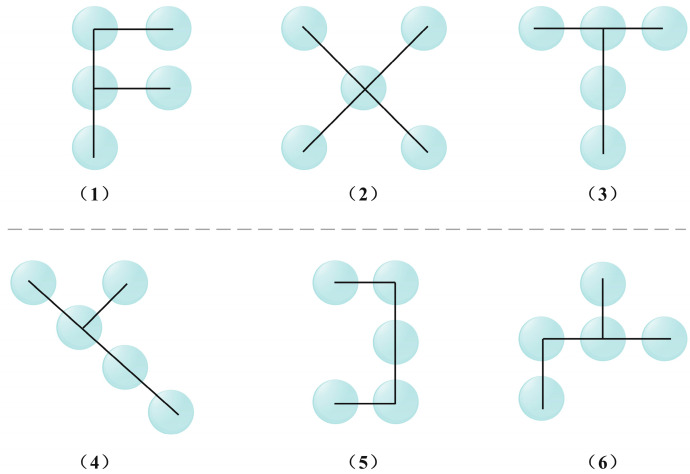
The 5 × 5 different initial sample shapes.

**Figure 12:**
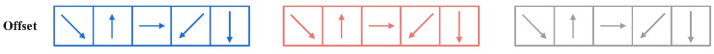
Offset adjusts the sample shape.

In AKConv, the design of convolution kernels has been innovatively improved to enhance adaptability and efficiency of convolutional networks. Unlike traditional convolution kernels, the size and shape of the kernels are not fixed but can be dynamically adjusted based on the density of lesions and disease characteristics to determine the number of parameters needed. Given the diverse types of diseases with varying lesion sizes and distributions, AKConv automatically adapts the size of convolution kernels during processing to effectively capture various lesion sizes and shapes, thereby enhancing feature extraction efficiency. The adaptive sampling shape is illustrated in Figure 10. Moreover, by designing different initial sampling shapes for a 5 × 5 sample grid, AKConv can accurately cover and process different image areas, leading to improved feature extraction accuracy, as shown in Figure 11. Additionally, AKConv can adjust the position of the convolution kernel using offsets to accommodate changes in local features at different locations, enabling better adaptation to non-rigid deformations, occlusions, and complex backgrounds in the target image. This capability provides a strong foundation for enhancing disease detection, as demonstrated in Figure 12.

## 3. Results and Discussion

### 3.1. Data Sets

**Figure 13:**
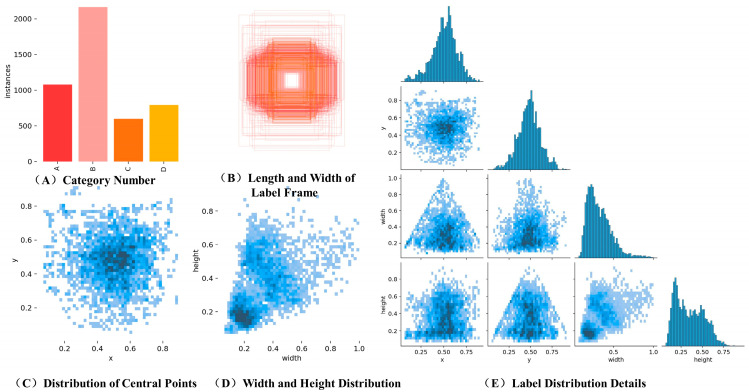
Dataset annotation file statistics and visualization.

The shooting scenes in this study are diverse, featuring various lighting conditions and weather changes. The background of the shooting environment is complex and includes a significant amount of interference information. The images have been captured using a Canon EOS 800D device, with a photo resolution of 4608 × 3456 pixels and saved in .PNG format. A total of 4650 images have been collected, encompassing four types of diseases: tea blight, tea white spot, tea sooty leaf disease, and tea ring spot. Among these, 3357 images with high-quality shooting effects have been annotated, with 2686 selected for training, 336 for verification, and 335 for testing. Labeling and visualization experiments have been conducted on the four disease types, with the results presented in Figure 13, each matrix unit represents the labels used during model training, and the color depth of the cells reflects the correlation between the corresponding labels. Dark cells indicate that the model has learned more strongly about the correlation between these two labels. Light colored cells indicate weak correlation. A represents the histogram of the number of categories in the data set; B indicates the length and width of each label frame after x and y values of all labels are set to the same position. C represents the distribution of x and y values in the image; D indicates the ratio of label width to label height in the data set; E represents the details of the label distribution in the original data set. The analysis reveals an uneven distribution of diseases within the self-built datasets. The positioning of the rectangular labeling boxes is precise, indicating the suitability of the proposed method for regional disease detection scenarios in Yunnan.

### 3.2. Experimental Environment and Parameter Setting

The experimental environment configuration and parameter settings are shown in Table 1.

This study evaluates the performance of the network model using parameters such as recall rate (Recall), precision rate (Precision), F1 balance score, multi-category average precision (mAP@0.5), detection speed, calculation amount, and other relevant metrics. FPS, the number of detection frames per second, is utilized to quantify the model’s detection speed. The specific calculation formula is shown in (27)–(31).

(27) $$Precision=\frac{T_{P}}{T_{P}+F_{P}}$$

(28) $$Recall=\frac{T_{P}}{T_{P}+F_{N}}$$

(29) $$F1=2\times \frac{Precision\times Recall}{Precision+Recall}$$

(30) $$AP=\int_{0}^{1}\;Precision\left(Recall\right)dRecall$$

(31) $$mAP=\frac{\sum_{i=1}^{C}AP(i)}{C}$$

In the above formula, $T_{P}$ represents the positive samples predicted by the model to be the positive class, $T_{N}$ represents the negative samples predicted by the model to be the negative class, $F_{P}$ represents the negative samples predicted by the model to be the positive class, and $F_{N}$ represents the positive samples predicted by the model to be the negative class.

### 3.3. Analysis of Model Training Results

**Figure 14:**
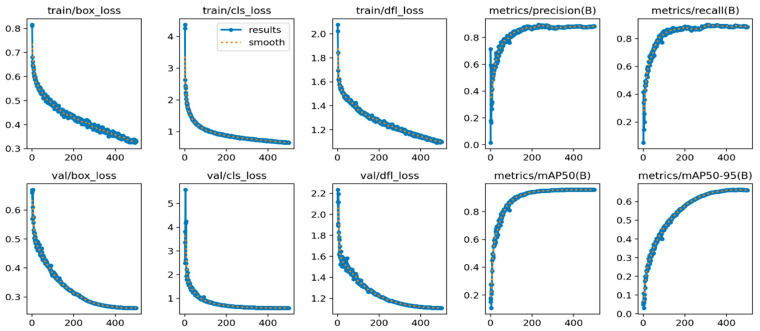
Evaluation results of YOLOv8-RMDA model training.

After 500 rounds of model training iterations, the convergence is approached after 450 rounds, yielding promising results on both the training and validation sets. Box_loss represents the mean inner-EIoU loss function, where a smaller value indicates higher detection prediction accuracy. Similarly, cls_loss denotes the mean classification loss function, with lower values indicating improved prediction accuracy. The dfl_loss, or free deformation loss, addresses target lesion shape issues in detection, with smaller values leading to better prediction outcomes. Performance degradation can occur due to changes in size. The mAP@0.5 and mAP@0.95 values reflect model prediction effectiveness, with higher values indicating better performance. The training and evaluation results of the YOLOv8-RMDA model can be observed in Figure 14.

### 3.4. Comparative Experiments

#### 3.4.1. Backbone Network Comparison Experiments

This study utilizes the enhanced YOLOv8 object detection network as the base model, incorporating an improved RFCBAM in place of commonly used lightweight feature extraction backbones like MobileNetV3 [37], MobileNetV2 [38], GhostNetV2 [39], and ShuffleNetV2 [40]. By maintaining consistent parameters except for the backbone network, the experimental results in Table 2 demonstrate the varying training effects of different backbone networks. The RFCBAM enhancement method exhibits superior training accuracy, recall rate, and average accuracy compared to MobileNetV3, MobileNetV2, GhostNetV2, and ShuffleNetV2. The mAP@0.5 shows an increase of 7.68%, 9.06%, 17.18%, and 6.57%, respectively, when compared to the other networks. Thus, enhancing the RFCBAM network leads to improved detection performance in the YOLOv8 model.

#### 3.4.2. Comparative Experiments with Different SPPF Structures

In order to assess the performance of the optimal SPPF, the enhanced MixSPPF is compared side by side with SPPF-DAattention, SPPF-LSA, and SPPF-LSKA [41,42]. The experimental results can be found in Table 3.

Table 3 illustrates that MixSPPF demonstrates superior performance in the mAP5@% metric. Among the tested models, SPPF-LSKA achieves the highest speed at 117 FPS, while SPPF-DAattention operates at the slowest speed of 186 FPS. MixSPPF operates at a speed of 145 FPS. Taking into account both mAP@0.5 and FPS, the optimized MixSPPF with a mixed pool emerges as the most favorable option.

#### 3.4.3. Comparative Experiments with Different Neck Network Feature Fusion Structures

The YOLOv8 network incorporates three distinct feature fusion structures: EfficientRepBiPAN, AFPN, and REPGFPN [43,44]. The experimental results can be found in Table 4.

Table 4 demonstrates that RepGFPN achieves an accuracy of 86.42% in mAP@0.5, surpassing EfficientRepBiPAN and AFPN. This suggests that RepGFPN excels in feature extraction. While EfficientRepBiPAN has the highest FPS in detection speed, its accuracy falls behind AFPN and RepGFPN. Consequently, the RepGFPN module is chosen to enhance the structure of the neck network.

For *ratio* in the inner-EIoU loss function, the results after taking the values of 0.75, 1, 1.25, and 1.5 comparisons are shown in Table 5.

When *ratio* = 1, the inner-EIoU loss function essentially becomes the EIoU loss function. The experimental results indicate that during the early and middle stages of tea growth, diseases are small targets that are difficult to distinguish. The labeling box is slightly offset, resulting in a low IoU. On the other hand, when *ratio* > 1, the auxiliary border is larger than the actual frame, which aids in IoU regression. Consequently, the experimental results when *ratio* > 1 are generally better than when *ratio* ≤ 1. However, the experimental results are suboptimal when *ratio* = 1.5. Therefore, the specific value of *ratio* should be adjusted and set according to the detection target of the experimental dataset. In this particular experiment, the specific value of *ratio* is set to 1.25.

#### 3.4.4. Ablation Experiments

The YOLOv8s model has been improved and the results of each improvement are statistically analysed, and the results are shown in Table 6.

In Table 6, A, B, C, and D represent the experimental results obtained by incorporating RFCBAM and the MixSPPF, Dynamic Head, and AKConv modules into the YOLOv8 model. The symbol √ indicates the addition of the module, while × indicates its absence. The experimental findings demonstrate that the model’s accuracy (P), recall (R), and average precision (mAP@0.5) have all shown improvement with the integration of each enhanced module. An analysis of the data in Table 6 reveals that combining A and B results in a 5.42% increase in recall rate (R) and a 3.15% increase in mAP without a decrease in FPS. Furthermore, the fusion of B, C, and D leads to an overall enhancement in the model’s performance, with accuracy rate (P), recall rate (R), and mAP@0.5 increasing by 2.32%, 6.92%, and 4.96%, respectively. Finally, the addition of module A on top of B, C, and D further optimizes the model, boosting the average accuracy (mAP@0.5) by an additional 1.01% while reducing computational costs. Despite this improvement, the FPS of 132 remains sufficient for real-time detection, enabling better detection of small targets in tea disease images.

#### 3.4.5. Comparative Experiments on the Performance of Different Network Models

To evaluate the efficacy of the enhanced YOLOv8 model, a comprehensive analysis has been conducted using a total of seven network models: Faster R-CNN, MobileNetV2, SSD, YOLOv5, YOLOv7, YOLOv8, and YOLOv8-RMDA. These models have been tested on custom datasets within identical training conditions. Performance evaluation indicators such as precision, recall, mAP@0.5, and FPS are used in this study. The experimental results are presented in Table 7, revealing that Faster R-CNN, MobileNetV2, and SSD exhibit subpar detection results for tea disease targets, with the highest average detection accuracy reaching only about 80%. On the other hand, the YOLOv8 and YOLOv8-RMDA models demonstrate superior detection performance. Specifically, the average precision rate of YOLOv8-RMDA is 20.41%, 17.92%, 12.18%, 12.18%, 10.85%, 7.32%, and 5.97% higher than Faster R-CNN, MobileNetV2, SSD, YOLOv5, YOLOv7, and YOLOv8, respectively. Moreover, the recall rate of YOLOv8-RMDA is 15.25% higher than that of the weakest Faster R-CNN model and 8.15% higher than the best-performing YOLOv8 model. In terms of FPS, YOLOv8-RMDA operates at 18, 19, and 15 frames lower than YOLOv5, YOLOv7, and YOLOv8, respectively. Notably, YOLOv8-RMDA exhibits a lower computational load, enabling improved real-time detection accuracy without significant amplitude changes, making it well-suited for regional scene detection applications.

**Figure 15:**
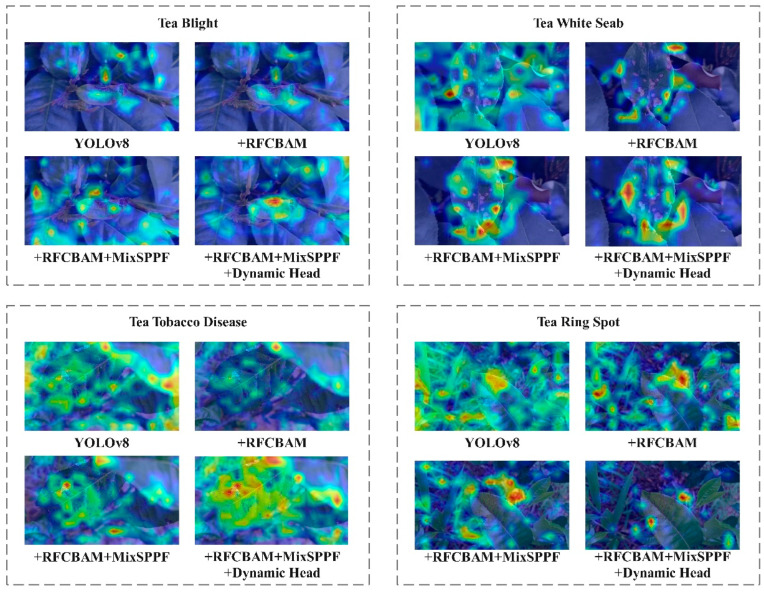
Comparison of heat maps of different module combinations of YOLOv8 model.

In this study, the YOLOv8-RMDA model’s capability to detect tea disease characteristics is further examined through the utilization of the Grad-CAM heat map analysis method. This method is employed to assess the effectiveness of various module combinations by visually displaying color changes from blue to red. The Grad-CAM heat map provides insights into whether the network model has successfully learned crucial features. The analysis focuses on representative tea disease images, with the results presented in Figure 15. The YOLOv8 network’s output heat map shows a lack of focus on the main disease area, with more attention given to irrelevant background areas. On the other hand, YOLOv8 + RFCBAM’s heat map displays scattered areas of concern, with higher weight around the disease but not yielding outstanding results. Moving on to YOLOv8 + RFCBAM + MixSPPF, the attention is concentrated on the tea disease’s characteristic area, with the disease’s characteristic color close to dark red, indicating higher responsiveness in that specific area. Finally, the YOLOv8 + RFCBAM + MixSPPF + Dynamic Head output heat map shows the darkest color in areas with severe tea disease, demonstrating a more concentrated focus on the disease itself and better identification performance in diseased areas.

**Figure 16:**
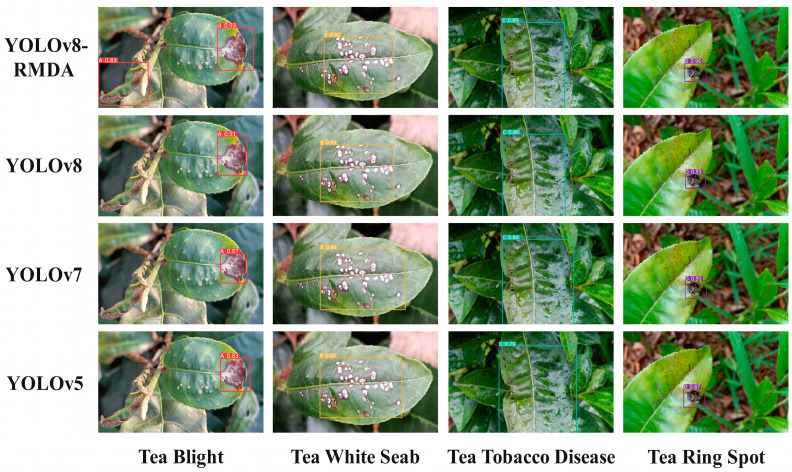
Comparison of detection effect of different models.

To further verify the improved detection performance of YOLOv8-RMDA in detecting different tea diseases in the complex environment of the Yunnan region, we selected 4 types of challenging tiny, dense diseases and tea disease images with similar backgrounds from the 335 images in the verification set for testing. We compared the performance of YOLOv8-RMDA with YOLOv8, YOLOv7, and YOLOv5 models to observe their confidence levels. The results are presented in Figure 16.

The results presented in Figure 16 demonstrate that the improved YOLOv8-RMDA model excels in detecting small, dense diseases and diseases with similar backgrounds. The confidence levels of the detection frames for the four diseases depicted in the image are notably higher at 93%, 92%, 89%, and 96%, respectively, compared to the YOLOv8, YOLOv7, and YOLOv5 models. Specifically, there is a 2%, 3%, 3%, and 3% improvement over the YOLOv8 model. Higher confidence in the detection frame indicates a greater likelihood of detecting the target in the prediction frame, resulting in more comprehensive details of the lesion target. YOLOv8-RMDA outperforms the YOLOv8, YOLOv7, and YOLOv5 models in terms of mAP@0.5, maintaining high accuracy while also reducing computational complexity to strike a balance between model weight and accuracy. Figure 16 compares the detection effects of different models.

## 4. Conclusions

This paper presents an enhanced YOLOv8-RMDA algorithm for the early detection of small targets related to tea diseases in complex scenes in Yunnan. The proposed algorithm addresses the issue of low recognition rates in traditional algorithms for small target detection tasks. The collection of regionally representative tea disease image datasets in Yunnan has been completed independently under natural conditions to ensure the authenticity and reliability of the experimental data. In the backbone, the RFCBAM and MixSPPF modules are introduced to enhance the C2f and traditional SPPF modules, reduce background environment interference, and improve the ability to extract global feature information. The experimental results demonstrate that utilizing the improved RFCBAM method yields significant advantages in precision and recall when compared to MobileNetV3, MobileNetV2, GhostNetV2, and ShuffleNetV2. The mAP@0.5 shows an improvement of 7.68% and 9.06%, respectively, over other networks. Additionally, the mAP@0.5 after implementing MixSPPF reaches 88.15%, which is 3.89% higher than the initial SPPF module. The use of feature fusion by RepGFPN in the neck region enhances the model’s ability to detect small target diseases. Although EfficientRepBiPAN achieves the highest FPS, its accuracy is lower than AFPN and RepGFPN. Furthermore, integrating the Dynamic Head detection head based on the previous improvement scheme enhances model accuracy. YOLOv8-RMDA outperforms Faster R-CNN, MobileNetV2, SSD, YOLOv5, YOLOv7, and YOLOv8 with an average accuracy increase of 20.41%, 17.92%, 12.18%, 12.18%, 10.85%, 7.32%, and 5.97%, respectively, effectively enhancing real-time detection accuracy. Finally, heat map analysis has been conducted on four prevalent tea diseases: tea leaf blight, tea white spot, tea coal leaf disease, and tea ring spot. The results indicate that the enhanced YOLOv8-RMDA outperforms YOLOv8 in terms of detection accuracy when the image target size is small, making it suitable for the early detection of small targets.

The next step involves expanding the tea disease image data set, establishing a model for multi-modal disease representation and visual recognition, and conducting research to enhance the model’s accuracy in recognizing small targets. This will enable more efficient agricultural work to be carried out.

## Figures and Tables

**Table 1:** Experimental environment configuration and parameter settings.

Configuration Items	Configuration Parameters
Computer operating system	Windows 11
CPU	Intel(R)CORE(TM)i7-11700
RAM	32 GB
GPU	NVIDIA GeForce RTX 3060
Compilation language Framework CUDA	Python 3.10.10 Pytorch 1.13.1 CUDA Version: 12.0
Epochs	500
Batch size	16

**Table 2:** Comparison of different lightweight feature extraction backbone networks.

Model	Backbone Network	P/%	R/%	mAP@0.5/%	FPS/S
YOLOv8	MobilenNetV3	71.86	72.38	80.34	177
YOLOv8	MobilenNetV2	69.32	67.88	78.96	179
YOLOv8	GhostNetV2	65.66	66.20	70.84	201
YOLOv8	ShuffleNetV2	77.81	75.30	81.45	156
YOLOv8	RFCBAM	78.42	83.26	88.02	149

**Table 3:** Comparative experiments of different SPPF structures.

Integration of Attention Mechanisms	P/%	R/%	mAP@0.5/%	FPS/S
SPPF	79.92	78.15	84.26	182
SPPF-DAattention	76.76	75.20	84.14	186
SPPF-LSA	79.48	80.40	87.78	181
SPPF-LSKA	78.42	80.38	88.06	117
MixSPPF	81.17	81.54	88.15	145

**Table 4:** Comparison of Different Feature Fusion Structures in Neck Networks.

Attention Mechanism	P/%	R/%	mAP@0.5/%	FPS/S
EfficientRepBiPAN	80.10	77.92	82.62	147
AFPN	76.94	78.62	84.76	160
RepGFPN	81.09	81.62	86.42	157

**Table 5:** Ablation experiments of *ratio*.

Ratio	P/%	R/%	mAP@0.5/%	FPS/S
0.75	72.52	73.10	77.46	145
1	77.68	74.45	78.28	140
1.25	79.36	80.47	79.88	139

**Table 6:** Ablation experiments.

Model	RFCBAM	MixSPPF	Dynamic Head	AKConv	P/%	R/%	mAP@0.5/%	FPS/S
YOLOv8	×	×	×	×	82.77	80.06	87.07	147
A	√	×	×	×	78.42	83.26	88.02	149
B	×	√	×	×	81.17	81.54	88.15	145
C	×	×	√	×	83.09	83.62	88.72	160
D	√	√	×	×	83.43	85.49	90.22	147
E	√	×	√	×	84.02	86.08	90.59	150
F	×	√	√	×	85.12	83.13	89.98	140
G	×	√	√	√	85.09	86.98	92.03	137
H	√	√	√	√	84.84	88.21	93.04	132

Note: √, use this algorithm; ×, do not use this algorithm.

**Table 7:** Comparison results of different network models for tea disease detection.

Model	P/%	R/%	mAP@0.5/%	FPS/S
Faster R-CNN	66.21	72.96	72.63	213
MobileNetV2	77.32	78.08	75.12	221
SSD	73.02	76.59	80.86	157
YOLOv5	82.37	80.39	82.19	150
YOLOv7	79.12	81.17	85.72	151
YOLOv8	82.77	80.06	87.07	147
YOLOv8-RMDA	84.84	88.21	93.04	132

## Data Availability

The data presented in this study are available upon request from the corresponding author (tli@ynu.edu.cn).
